# Bioactive Compounds Produced by *Hypoxylon fragiforme* against *Staphylococcus aureus* Biofilms

**DOI:** 10.3390/microorganisms5040080

**Published:** 2017-12-12

**Authors:** Kamila Tomoko Yuyama, Clara Chepkirui, Lucile Wendt, Diana Fortkamp, Marc Stadler, Wolf-Rainer Abraham

**Affiliations:** 1 Chemical Microbiology, Helmholtz Centre for Infection Research (HZI), Inhoffenstraße 7, 38124 Braunschweig, Germany; kamila.yuyama@helmholtz-hzi.de (K.T.Y.); diana.fortkamp@helmholtz-hzi.de (D.F.); 2 Microbial Drugs, Helmholtz Centre for Infection Research (HZI), Inhoffenstraße 7, 38124 Braunschweig, Germany; clara.chepkirui@helmholtz-hzi.de (C.C.); lucile.wendt@helmholtz-hzi.de (L.W.); marc.stadler@helmholtz-hzi.de (M.S.); 3 Department of Exact Sciences, Escola Superior de Agricultura “Luiz de Queiroz” (ESALQ), Piracicaba, SP 13418-900, Brazil

**Keywords:** biofilm dispersion, *Staphylococcus aureus*, *Hypoxylon fragiforme*, secondary metabolites

## Abstract

Treating infections organized in biofilms is a challenge due to the resistance of the pathogens against antibiotics and host immune cells. Many fungi grow in a wet environment, favorable for the growth of bacterial biofilms, and we speculated that fungi possess some strategies to control these bacterial biofilms. A fungus identified as *Hypoxylon fragiforme*, was collected in the Harz Mountains, Germany, and its mycelial culture was fermented in different culture media for 67 days to test its biological potential against bacterial biofilms. Sclerin, sclerin diacid and its 3-methyl monoester (methyl 1-(5-hydroxy-6-carboxylic-2,3,4-trimethylphenyl) propionate) are here described for the first time from this fungus. Sclerin and its diacid interfered with the biofilm formation of the pathogen *Staphylococcus aureus*, inhibiting 86% and 80% of the biofilm at 256 μg mL^−1^, respectively, but not killing the bacterium. Interestingly, the monomethylester of sclerin diacid was inactive. Although these compounds did not possess any activity against a pre-formed biofilm, they prevented its formation at subtoxic concentrations. Furthermore, sclerin and its diacid displayed a high specificity against *Staphylococcus aureus*, indicating a good strategy against pathogenic biofilms when combined with antibiotics.

## 1. Introduction

Biofilms are structured microbial communities where microorganisms are embedded in an extracellular complex matrix of polymeric substances and they can adhere to an inert or living surface [1,2]. In nature, 99% of bacteria aggregate as biofilms [3], which are produced when bacteria reproduce vertically and horizontally on a surface to form a multicellular, sessile colony, that secretes a matrix of polysaccharides, protein, and extracellular DNA, allowing them to form micro-niches and maintain steep chemical gradients [4,5].

Although biofilms play a protective role at the skin and mucosa, many others, e.g., in wounds, on implants or teeth are really harmful [6]. According to the National Institute of Health more than 80% of human bacterial infections are associated with biofilms, e.g., in the catheter-associated urinary tract infection [7], in contact lenses [8], in the cystic fibrosis lung [9] or in the gastric mucosa. The main challenge in controlling biofilms is its protection of the bacterial cells against antibiotics and the host immune system caused by the biofilm lifestyle.

One important step to form the biofilm is the communication, called quorum sensing, between the microorganisms through small molecules (autoinducers) that play an important role in coordinating bacterial virulence [10]. Blocking quorum sensing is a good strategy to combat the biofilm formation or to disperse existing ones [11]. Some studies have shown that fungi synthesize compounds that interfered with the biofilm formation of pathogenic bacteria [12,13]. It has been hypothesized that fungi have some strategies to protect themselves against bacterial biofilm formation on their fruiting bodies, since fungi are exposed to a wet climate, which is favorable for biofilm formation.

*Hypoxylon fragiforme* is the type species of the family Hypoxylaceae, which was recently resurrected and emended to accommodate the genera that have stromatal pigments and a nodulisporium-like asexal state and were previously included in the Xylariaceae [14,15]. A previous study has revealed that this species is able to produce various types of secondary metabolites, and its metabolite profiles change drastically during ontogeny of the stromata [16]. Although several stromatal secondary metabolites like cytochalasins [17] and azaphilones show many biological activities, not much is known about the ability of compounds produced by this fungus in different culture media to inhibit biofilm formation. The aim of this work was therefore to investigate the potential of secondary metabolites produced by *H. fragiforme* in different culture media against pathogenic biofilms.

## 2. Materials and Methods

### 2.1. Reagents

Acetonitrile, chloroform, ethyl acetate and methanol were purchased from J.T.Baker (München, Germany) respectively, d-chloroform, formic acid 98%, Potato Dextrose (PD), Luria-Bertani broth (LB), sodium chloride (NaCl), potassium chloride (KCl), potassium dihydrogen phosphate (KH_2_PO_4_), d-methanol, and trifluoroacetic acid (TFA) from Carl Roth GmbH (Karlsruhe, Germany). Bacto malt extract, Bacto peptone and agar were from BD (La Point de Claix, France), d-glucose from Merck (Darmstadt, Germany). Disodium hydrogen phosphate (Na_2_HPO_4_) was purchased from J.T.Baker^®^ (The Netherlands), crystal violet from Fluka (Steinheim, Germany), tetracycline and potato dextrose agar (PDA) from Sigma Aldrich (Taufkirchen, Germany) respectively.

### 2.2. Microorganisms

*Bacillus cereus* DSM 626, *Staphylococcus epidermidis* ATCC 35984, *Streptococcus mutans* UA59, *Staphylococcus aureus* DSM 1104 were purchased from the German Collection of Microorganisms and Cell Cultures (DSMZ, Braunschweig, Germany). *Pseudomonas aeruginosa* PA14, *Escherichia coli* MT102 and the other bacteria were applied in antimicrobial and antibiofilm assays. Bacterial strains were maintained on LB agar at 4 °C.

### 2.3. Isolation and Fungal Identification

The fungus was collected on a decayed trunk of *Fagus* sp. in the Harz mountains (latitude 51°45′21′′, longitude 10°32′17′′, 724 m), in Germany and identified by macroscopical and microscopical characteristics. A culture of the fungus was obtained by spore isolation on PD agar (PDA) and the authenticity of the culture was verified by molecular data. For morphological characterization, the stromatal surface, the extraction of stromatal pigments in 10% KOH, and the size and shape of the ascospores were analyzed. For molecular identification, DNA was extracted with the NucleoSpin Plant II kit (Macherey-Nagel, Düren, Germany) and the internal transcribed spacer region of the ribosomal RNA gene region (ITS) and a partial region of the beta-tubulin gene region (TUB2) were amplified using the fungal primers (ITS1F/ITS4 [18,19] and T1/T2/T21 [20]) and PCR protocols as described in [14]. PCR products were purified using PCR purification Kit (BioBasic Inc., Toronto, ON, Canada), sequenced by the In-House sequencing service of the HZI (GMAK) and by Eurofins (Ebersberg, Germany) aligned using Sequencher 4.10.1, and compared to the respective sequences of the closest related species using MEGA 6 [21]. Sequences were deposited in GenBank with the accession numbers (MG021164-ITS, MG231903-TUB2).

### 2.4. Fermentation

Mycelia pellets (5 × 5 mm) of the isolate grown on malt extract agar (3% malt extract, 0.5% Bacto peptone and 1.5% agar) and potato dextrose agar (PDA) were transferred to 2 L Erlenmeyer flasks containing 1 L of malt extract (ME) broth (3% malt extract, 0.5% bacto peptone), PD, rice (Kaufland, Braunschweig, Germany) and minimal medium (MM) described by [22]. The fungus was static incubated during 67 days at 22 °C in the dark; this time was necessary for the fungus to grow, to produce spores, to consume the glucose available and to secrete some visible secondary metabolites, like the green pigment hypoxyxylerone [23]. After that, the broth was filtrated, extracted with ethyl acetate and tested for antibiofilm activities.

### 2.5. Inhibition of Biofilm Formation

A pre-inoculum of *S. aureus* was adjusted to reach the turbidity of a 0.5 McFarland standard and 150 μL of CASO with 4% glucose with pH 7.0 was added together with the serial diluted compounds (256–8 μg mL^−1^) and incubated in 96-well tissue microtiter plates (TPP, Trasadingen, Switzerland) for 20 h at 37 °C. Methanol was used as negative control. Subsequently, the plates were stained with crystal violet and measured by a plate reader, following the protocol [24]. All experiments were made in triplicates with two repetitions. For *S. epidermidis*, *B. cereus*, *E. coli* and *S. mutans*, the same protocol was used, but they were cultivated in LB medium and incubated in 96-well no tissue microtiter plates (Falcon Micro Test^TM^, Tewksbury, MA, USA). Only *P. aeruginosa* was cultivated in CASO.

The determination of the minimal inhibitory concentration (MIC) was done following the protocol of [12]. The respective culture media and methanol were used as negative control; tetracycline (100 μg mL^−1^) was the positive control.

### 2.6. Dispersion of Pre-Formed Biofilms

The dispersion of the pre-formed biofilm of *S. aureus* was determined as described above with the following changes. After 24 h of incubation, the supernatant was removed from the wells, the wells were washed with phosphate-buffered saline (PBS, 0.8% NaCl, 0.02% KCl, 0.14% Na_2_HPO_4_, 0.02% KH_2_PO_4_) and 150 μL of CASO with 4% glucose was added together with the serial diluted compounds (256–8 μg mL^−1^). Staining of the biofilm, replicates and controls were described as for the biofilm inhibition.

### 2.7. Cytotoxicity Assay

To check the cytotoxicity the compounds (**2**) and (**3**) were tested against L-929 mouse fibroblasts and HeLa (KB-3.1) cell lines. The cell lines were cultivated in DMEM (GIBCO BRL, Schwerte, Germany), high glucose medium supplemented with 10% fetal calf serum (GIBCO BRL, Germany), at 37 °C under 10% CO_2_. To execute the test, 60 μL of serial dilutions from an initial stock of 1 mg mL^−1^ in methanol of (**2**) and (**3**) were added into 120 μL of suspended cells (50,000 mL^−1^) in 96-well microtiter plates. After 5 days of incubation, the growth inhibition (IC_50_) was determined using an MTT assay [25]. Epothilon B (1 μg mL^−1^) was used as positive control.

### 2.8. Purification and Structure Elucidation of Compounds

The crude extracts were fractionated by preparative LC (HPLC 2020, Gilson, Middleton, WI, USA) equipped with a VP Nucleodur 100-7 C18 ec column (125 × 40 mm, 7 μm; Macherey-Nagel, Düren, Germany). Mobile phase: solvent A: H_2_O (Milli-Q, Millipore, Schwalbach, Germany) with 0.05% TFA; solvent B: acetonitrile, 0.05% TFA. The elution gradient used was: (i) started with 10 to 70% of solvent B during 45 min; (ii) gradient shift from 70 to 100% of solvent B during 5 min; (iii) isocratic condition of 100% solvent B for 5 min. UV detection was carried out at λ 210, 254 and 350 nm.

The compounds (**2**)–(**4**) were purified by reverse phase LC (solvent A/solvent B). The elution gradient used was: (i) started with 48 to 60% of solvent B for 30 min; (ii) gradient shift from 90 to 100% during 3 min and (iii) isocratic condition of 100% solvent B for 5 min with preparative (Kromasil) 250 × 20 mm 7 μL C-18 column as stationary phase. To purify (**1**), the same conditions were applied, only the gradient was different, starting with 38 to 45% of solvent B during 25 min.

The identity of the compounds was confirmed by High Resolution Electrospray Ionisation Mass Spectrometry (HR-ESIMS), using the same instrumentals setting of [25]. NMR spectra were recorded on a Bruker Ascend 700 spectrometer with 5 mm TXI cryoprobe (^1^H 700 MHz, ^13^C 175 MHz) and Bruker AV II-600 (^1^H 600 MHz, ^13^C 150 MHz) spectrometers [26].

1-Methyl-(5-hydroxy-6-carboxylic-2,3,4-trimethylphenyl) propionate methylester (**4**): Retention time RT 9.76 min; UV: λ_max_ 215, 258, 320 nm; MS: 267.1402 ([M + H]^+^, 267.1233 calc. for C_14_H_19_O_5_), 235.1117 [M + H-MeOH]^+^, 221.1316, 207.1154, 189.1036; NMR see Table 1.

## 3. Results

Following the *Hypoxylon* spp. key proposed by Fournier et al. [27], the morphological features of our strain were similar to *H. fragiforme*, the other closest species *H. howeanum* was excluded, because of its bigger ascospores (12–6 μm). In addition, a comparison of the ITS and TUB2 sequence of the studied specimen with the available sequences in GenBank showed high identity (99%) with *H. fragiforme* (closest hit in GenBank: KC477229(ITS), KX271282(TUB2)). Based on morphological and molecular characteristics, the specimen was identified as *H. fragiforme*.

We analyzed the production of secondary metabolites in four different culture media (PD, ME, MM and rice). In all culture media, iso-ochracein (**1**) was formed. In MM and in rice media, cytochalasins were detected but not in PD and ME. Instead, the fungus produced in the latter two media compounds (**2**) and (**3**), which were to the best of our knowledge never before reported from this species.

**Figure 1:**
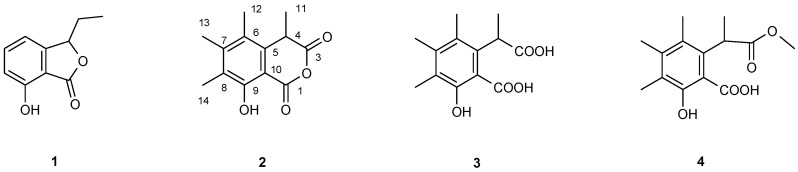
Iso-ochracein (**1**), sclerin (**2**), sclerin diacid (**3**), and its 3-methyl monoester methyl 1-(5-hydroxy-6-carboxylic-2,3,4-trimethylphenyl) propionate (**4**) were isolated from the culture broth of *Hypoxylon fragiformis*. Compounds (**2**) and (**3**) inhibited the formation of biofilms from the pathogen *Staphylococcus aureus* but compounds (**1**) and (**4**) were inactive.

Compound (**1**) with a yield of 11.6 mg L^−1^ had a [M + H]^+^ ion of 179.0710 which fits to the sum formula of C_10_H_10_O_3_ for the secondary metabolite. ^1^H NMR spectra showed a 1,2,3-trisubstituted aromatic ring. Various homo- and heteronuclear 2D NMR spectra led to the identification of (**1**) as iso-ochracein, first reported from *H. fragiforme* [28] (Figure 1). In HR-ESI-MS the second compound had a [M + H]^+^ ion of 235.0970 which is fulfilled by C_13_H_14_O_4_. ^1^H NMR spectra showed three aromatic methyl groups at δ_H_ = 2.189, 2.252 and 2.306, a methyl doublet at δ_H_ = 1.570 (*J* = 7.4 Hz) and a broad singlet at δ_H_ = 10.754 (Figure S1). Again, 2D NMR spectra, especially heteronuclear multiple-quantum correlation (HMQC) NMR spetra, could supply the connections between these signals and resulted in the identification of compound (**2**) as sclerin [29] yielding 4.7 mg L^−1^. The obtained NMR data fitted well to those reported in the literature. Sclerin was first reported from *Sclerotinia sclerotiorum* [30] but never from *Hypoxylon fragiforme*.

Compound (**3**) showed ^1^H NMR signals which were very similar to those of sclerin (Figure S2). Its molecular ion, however, was 18 Da heavier than sclerin. Extensive NMR studies similar to the ones applied to the identification of sclerin finally led to the identification of (**3**) as the free diacid of sclerin [31] with a yield of 8.5 mg L^−1^. Among other differences in the ^13^C-NMR spectra, a significant low-field shift of C-1, C-3 and C-4 compared to those of sclerin due to the opening of the anhydride could be seen (Table 1). Finally, compound (**4**) with a yield of 4.1 mg L^−1^ displayed in ^1^H NMR again resonances of three aromatic methyl groups, a methyl doublet and in addition a methoxy group (Figure S3, Table 1). Its structure could be deduced from ^1^H,^1^H- and ^1^H,^13^C-NMR spectra. The position of the methoxy group was found to be at C-3 because of its ^1^H,^13^C-long-range coupling to C-4, δ_C_ = 42.22. This was corroborated in the ^13^C-NMR spectrum by a low-field shift of C-1 but a high-field shift of C-3 due to the methyl group at C-3 compared to those of (**3**). Interestingly, the 1-methyl ester of (**3**) could not be detected. Starratt and Lazarovits [32] mentioned in 1996 both the 1- and the 3-methyl monoester of diacid (**3**) when treating sclerin (**2**) with methanol under acidic conditions. However, neither physical data nor NMR data have been published [32]. Careful analysis of the extract revealed that compound (**4**) was formed by methanolysis during chromatography identifying it as an artefact. Both ^1^H and ^13^C NMR data and the bioactivity of this compound were first reported here.

**Figure 2:**
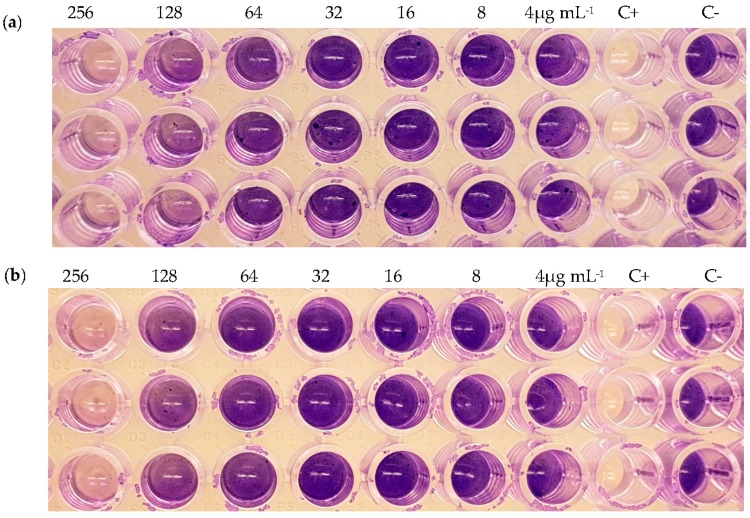
Inhibition of the biofilm formation from *S. aureus* of compound (**2**) (**a**) and (**3**) (**b**). Each column has three replicates in different concentrations of the compounds (256, 128, 64, 32, 16 and 8 μg mL^−1^) and their respectively controls. C+: Positive control with tetracycline (100 μg mL^−1^). C−: Negative control with methanol 3%.

While none of the purified compounds displayed neither any antibacterial activities against *S. aureus* nor dispersed its pre-formed biofilms at a minimal inhibitory concentration (MIC) lower than 256 μg mL^−1^, compounds (**2**) and (**3**) showed an inhibition of biofilm formation of 86% and 80% at 256 μg mL^−1^ (Figure 2). The fact that these compounds interfered with biofilm formation but were not toxic at this concentration for planktonic cells points to an inhibition of some essential component of quorum sensing (Table 2).

In addition, compounds (**2**) and (**3**) have a high specificity against *S. aureus*, because these compounds did not affect biofilm formation of the other tested bacteria (Table 2). Compound (**3**) and (**4**) are derivatives of sclerin (**2**), but only sclerin and compound (**3**) possessed good activity against biofilms and this is the first biological activity described for compound (**3**). Although, compounds (**1**) and (**4**) had no activity against the biofilm, (**1**) affected the growth of *E. coli* at 256 μg mL^−1^, decreasing half of the optical density (O.D.) compared with the control after 18 h of growing. The halfester (**4**) was synthesized by this fungus and is described here for the first time. None of the bioactive compounds displayed any cytotoxicity against mammalian cells (L-929 and HeLa (KB-3.1)) until the maximum concentration range (1000 μg mL^−1^).

## 4. Discussion

While *H. fragiforme* has the ability to synthesize KOH extractable stromatal pigments [33] and a lot of stromatal metabolites [34], its cultures has different metabolic products. All the cultures tested produced iso-ochracein, as reported [35], and the green pigment hypoxyxylerone, an inhibitor of topoisomerase I [35,36]. Some cytochalasins (H and L-696,474) reported in [16,37] were also produced in MM and rice media, but not in PD and ME.

Sclerin (**2**), its diacid (**3**) and its 3-methyl monoester (**4**) were produced only in PD and ME cultures and for the first time reported in *H. fragiforme*. Sclerin (**2**) has been known for several decades; it is produced by the fungi *S. sclerotiorum* [30] and *Aspergillus carneus* [38]. Many biological activities were previously reported for sclerin (**2**), such as promotion of lipase formation and enhanced growth of various plant seedlings [30], enhancement of the production of aminoglycoside antibiotics from *Streptomyces kanamyceticus, S. ribosidificus* and *S. griseus* [39], maintenance of energy linked functions in plant and rat liver mitochondria during aging [40] and toxicity against three cruciferous species.

The diacid (**3**) is a derivative of sclerin (**2**), and it is formed in aqueous or alcoholic environments, where the anhydride ring of sclerin is cleaved [32]. No biological activities of (**3**) were reported, albeit in this paper it is presented that both sclerin (**2**) and its diacid (**3**) inhibit the formation of *S. aureus* biofilms at subtoxic concentrations, similar to the activity of aurantiogliocladin [12]. While the free diacid (**3**) is active against *S. aureus* biofilms, its 3-monomethyl ester (**4**) is not. These results may be explained by sclerin (**2**) as the active compound which is probably in equilibrium with the open diacid (**3**) but not formed from the monoester (**4**) under physiological conditions. However, none of these compounds could disperse pre-formed biofilms, probably because this seems to be an independent mechanism [1].

Sclerin (**2**) and its diacid (**3**) displayed high specificity against *S. aureus*, and they had no effect against other bacteria tested, including the closely related *S. epidermidis*. The bioactivities of both compounds were rather low but significant inhibitions of *S. aureus* biofilms could be seen at concentrations well below the MIC and the LD_50_. However, using higher active chemical derivatives of (**2**) or (**3**) combined with an antibiotic could be a promising strategy to reduce specific staphylococci infections, since the bioactive compounds even in higher concentrations (1000 μg mL^−1^) did not display cytotoxicity against mammalian cells. Many studies demonstrated the efficacy of the use of quorum-quenching compounds together with antibiotics [41,42].

## 5. Conclusions

Our working hypothesis that many fungi developed strategies for the protection against biofilm infections was confirmed for *Hypoxylon fragiforme*. Although none of the four compounds (iso-ochracein (**1**), sclerin (**2**), sclerin diacid (**3**) and its 3-methyl ester (**4**)) showed antibiotic activities, compounds (**2**) and (**3**) could affect the formation of biofilms. This ability was not directed against all bacteria but found only for Gram-positive bacteria and here, especially for *Staphylococcus aureus*. The mechanism of action was not elucidated but from the fact that sclerin (**2**) and its open diacid (**3**) were active but the monomethyl ester of (**3**) was not; this points to the anhydride being the essential moiety for this activity. Although the activity of the compounds was not high, the total lack of cytotoxicity in higher concentration (1000 μg mL^−1^) and the possibility of combination of more active synthetic derivatives with antibiotics may be relevant in the future control of the pathogen *Staphylococcus aureus* [43].

## Figures and Tables

**Table 1:** NMR data of compounds (**2**)–(**4**).

	(2)		(3)		(4)	
	^1^H	^13^C	^1^H	^13^C	^1^H	^13^C
C-1	-	166.22	-	172.94		179.91
C-3	-	168.68	-	176.36		171.40
C-4	4.160 (1H, q, *J* = 7.4 Hz)	38.65	4.468 (1H, q, *J* = 7.4 Hz)	42.01	4.315 (1H, q, *J* = 7.6 Hz)	42.22
C-5	-	134.53	-	137.43		136.17
C-6	-	123.89	-	126.39		123.88
C-7	-	147.70	-	142.84		143.40
C-8	-	124.61	-	123.56		126.75
C-9	10.754 (1H, s) ^1^	158.82	7.487 (1H, s)	157.54	10.767 (1H, s)	157.76
C-10	-	101.41	-	109.98		109.52
C-11	1.570 (3H, d, *J* = 7.4 Hz)	22.18	1.537 (3H, d, *J* = 7.4 Hz)	17.12	1.526 (3H, d, *J* = 7.6 Hz)	22.10
C-12	2.306 (s)	17.38	2.233 (s)	16.61	2.240 (s)	16.70
C-13	2.189 (s)	14.40	2.125 (s)	15.94	2.283 (s)	16.83
C-14	2.252 (s)	11.83	2.189 (s)	11.91	2.240 (s)	12.38
OMe	-	-	-	-	3.871 (s)	51.36

^1^ OH at C-9.

**Table 2:** Antimicrobial MIC and biofilm inhibition.

Strain	Compound	MIC (μg mL^−1^)	Inhibition of Biofilm Formation (%)	Inhibition of Pre-Formed Biofilm (%)
*S. aureus*	(**1**)	>256	- ^2^	-
	(**2**)	>256	86 (256 μg mL^−1^) 51 (128 μg mL^−1^)	-
	(**3**)	>256	80 (256 μg mL^−1^) 34 (128 μg mL^−1^)	-
	(**4**)	>256	-	-
*E. coli*	T ^1^	>256	-	nt ^3^
*B. cereus*	T	>256	-	nt
*S. mutans*	T	>256	-	nt
*S. epidermidis*	T	>256	-	nt
*P. aeruginosa*	T	>256	-	nt

^1^ (T) all compounds; ^2^ (-) no activity; ^3^ (nt) not tested.

## Supplementary Materials

The following are available online at www.mdpi.com/2076-2607/5/4/80/s1.
