# Supplementary material for: Bioactive Compounds Produced by Hypoxylon fragiforme against Staphylococcus aureus Biofilms
Source: Microorganisms. 2017 Dec 12;5(4):80. doi: 10.3390/microorganisms5040080 (PMC5748589; doi:10.3390/microorganisms5040080)
Supplement: Supplementary file 1 [file microorganisms-05-00080-s001.pdf]

Supplement material of

Bioactive compounds produced by *Hypoxylon fragiforme* against *Staphylococcus aureus* biofilms

Kamila Tomoko Yuyama[1], Clara Chepkirui[2], Lucile Wendt[2], Diana Fortkamp[1,3], Marc Stadler[2], Wolf-Rainer Abraham[1,*]

1 Helmholtz Centre for Infection Research (HZI), Chemical Microbiology, Inhoffenstraße 7, 38124 Braunschweig, Germany; kamila.yuyama@helmholtz-hzi.de, diana.fortkamp@helmholtz-hzi.de
2 Helmholtz Centre for Infection Research (HZI), Microbial Drugs; clara.chepkirui@helmholtz-hzi.de, lucile.wendt@helmholtz-hzi.de, marc.stadler@helmholtz-hzi.de
3 Escola Superior de Agricultura "Luiz de Queiroz" (ESALQ), Department of Exact Sciences, Piracicaba - SP, Brazil; dianafortkamp@usp.br
* Correspondence: wolf-rainer.abraham@helmholtz-hzi.de; Tel.: +49-531-6181-4300

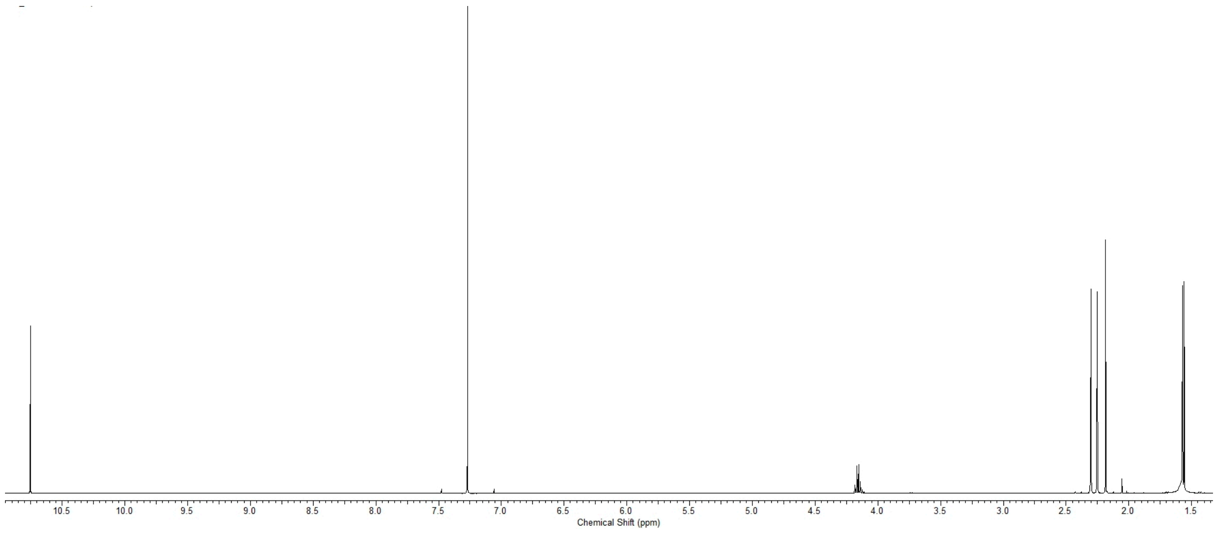

Figure S1: ^{1}H NMR spectrum of sclerin (**2**) in $CDCl_3$

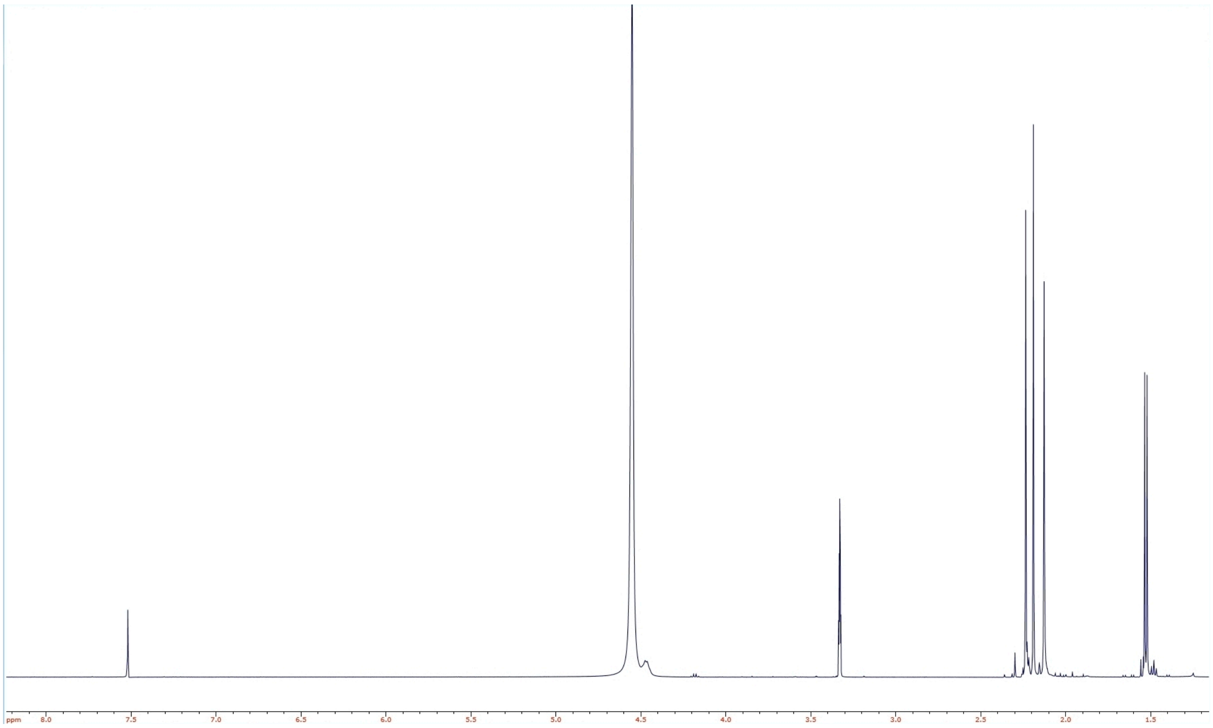

Figure S2: ^{1}H NMR of the diacid of sclerin (**3**) in d6-methanol

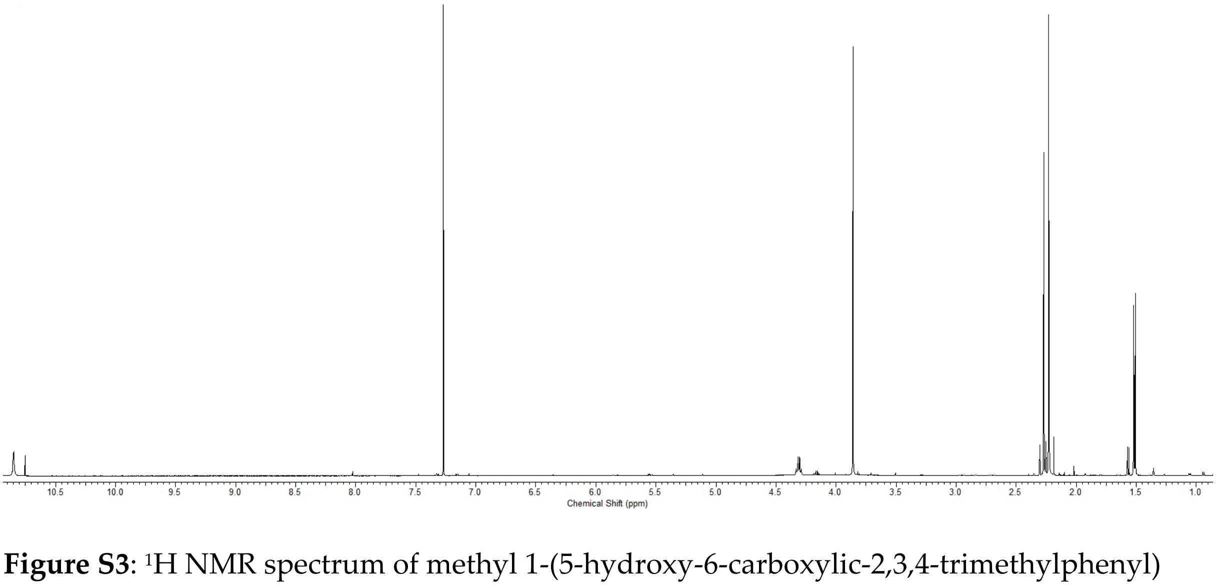

Figure S3: ^{1}H NMR spectrum of methyl 1-(5-hydroxy-6-carboxylic-2,3,4-trimethylphenyl) propionate (**4**) in $CDCl_3$
